# An Analysis of Information Sources of YouTube Videos Pertaining to Tattoo Removal: Cross-sectional Study

**DOI:** 10.2196/42759

**Published:** 2022-10-25

**Authors:** Benjamin Gallo Marin, Ogechi Ezemma, Fabio Stefano Frech, Julio C Flores Servin, Ben S Rhee, Kathleen M Mulligan, Katie A O' Connell, Isabelle Moseley, Carlos G Wambier

**Affiliations:** 1 Department of Dermatology Warren Alpert Medical School of Brown University Providence, RI United States; 2 Department of Dermatology Miller School of Medicine University of Miami Miami, FL United States; 3 Department of Dermatology Harvard Medical School Boston, MA United States; 4 Department of Dermatology Case Western Reserve University School of Medicine Cleveland, OH United States; 5 Department of Medicine Vanderbilt University School of Medicine Nashville, TN United States

**Keywords:** tattoo, tattoo removal, laser, internet, YouTube, misinformation, Food and Drug Administration, FDA, professional information, digital, research, skin, skin care, skincare, care, consultation, safe, evidence, dermatologist

## Abstract

**Background:**

The American Academy of Dermatology and the Food and Drug Administration recommend consultation with a dermatologist prior to undergoing laser tattoo removal. However, non–health care professionals offer tattoo removal. Understanding the information available on the internet for patients regarding tattoo removal is important given that individuals are increasingly consulting digital sources to make decisions regarding skin care. Prior research has identified that YouTube contains misinformation on dermatologic health.

**Objective:**

Here, we present a cross-sectional study that determined the sources of information in YouTube videos that discuss tattoo removal and described the content presented to viewers.

**Methods:**

Using the query “tattoo removal,” we reviewed English-language YouTube videos that explicitly discussed tattoo removal. The following data were recorded: profession of the presenter, tattoo removal method discussed, whether an explicit recommendation to see a dermatologist or physician was present in the video, and number of views.

**Results:**

We analyzed 162 YouTube videos. We found that the majority were presented by non–health care professionals (n=125, 77%), with only 4 (3.7%) records of this subset recommending viewers to seek consultation from a dermatologist to ensure safe and adequate tattoo removal.

**Conclusions:**

Based on our findings, we recommend that dermatologists and other health care professionals provide high-quality, evidence-based information to viewers on tattoo removal and encourage dermatology societies to share via their social media platforms information about the importance of consulting a dermatologist for tattoo removal.

## Introduction

Laser-based technologies are the preferred methods for tattoo removal, and the American Academy of Dermatology and the Food and Drug Administration recommend consultation with a dermatologist prior to undergoing these procedures [1]. However, tattoo removal performed by non–health care professionals and via do-it-yourself methods (eg, scrubs, at-home lasers) are widely advertised [2]. Inadequate tattoo removal may lead to dermatologic complications, including scarring and suboptimal cosmetic outcomes. The free video platform YouTube is often accessed by individuals seeking information on cosmetic procedures. However, prior research has shown that YouTube contains misinformation regarding skin health [3-6]. This study aimed to determine the sources of information of YouTube videos discussing tattoo removal and to describe the contents that viewers are exposed to. We hypothesized that most YouTube videos pertaining to tattoo removal are presented by non–health care professionals, with many videos failing to recommend viewers to seek consultation with a dermatologist for these procedures.

## Methods

A YouTube query for “tattoo removal” was performed on June 22, 2022. To mitigate selection bias, the search was conducted using incognito mode. Eligible videos were presented in English, featured audio (ie, rather than text-only), and explicitly discussed tattoo removal. Videos that met the inclusion criteria were then independently analyzed by 2 researchers, and the following variables were recorded: profession of the presenter, tattoo removal method discussed, whether an explicit recommendation to see a dermatologist or physician was present in the video, and number of views.

## Results

A total of 186 videos were initially identified. After excluding videos unrelated to tattoo removal, without audio, or not in English, we included 162 (87%) of these records in our analysis. Of these 162 videos, most videos were presented by non–health care workers (n=125, 77%), with only 37 (23%) featuring health care professionals (ie, either voice-over or on-screen). Among health care professionals, presenters included dermatologists (n=27, 73%), registered nurses (n=5, 14%), plastic surgeons (n=3, 8%), and physician assistants (n=2, 5%). Laser removal was the most common tattoo removal method discussed across all videos (n=143, 88%); 35 videos from health care professionals addressed this approach, and none of them provided a discussion of technical parameters such as laser settings. The remaining 2 videos created by health care professionals discussed excisional surgery and the ineffectiveness of salt and cocoa butter scrubs. All videos presented by health care professionals suggested that viewers seek tattoo removal through physicians, with treatment in a dermatology office (n=33, 89%) being the most frequent recommendation (Table 1).

**Table 1:** Presenters and methods in YouTube videos discussing tattoo removal included in this study (N=162).

Presenters and methods		Videos, n (%)
**Health care professionals**		37 (23)
	Dermatologists	27 (73)
	Plastic surgeon	3 (8)
	Registered nurses	5 (14)
	Physician assistants	2 (5)
**Health care professional methods**		
	Laser	35 (95)
	Excisional surgery	1 (3)
	Cocoa butter scrub (comment on lack of effectiveness)	1 (3)
Non–health care workers		125 (77)
**Non–health care worker methods**		
	Laser	108 (86)
	Microneedle patch	1 (0.8)
	Removal cream	1 (0.8)
	Lemon juice scrub	6 (5)
	Yogurt scrub	1 (0.8)
	Salt scrub	6 (5)
	Oral herb therapy	1 (0.8)
	Ink and light	1 (0.8)

While 108 videos by non–health care professionals discussed lasers, only 4 (3.7%) explicitly stated that viewers should schedule a consultation with a dermatologist to discuss the removal of their tattoos. The remaining videos focused on the presenters’ experiences visiting laser clinics (n=95) or utilizing lasers at home (n=9). Nonlaser methods discussed in the videos presented by non–health care professionals included the use of scrubs composed of lemon juice (n=6), salts (n=6), and yogurt (n=1); microneedle patches (n=1); creams (n=1); oral herb therapy (n=1); and the application of ink followed by light (n=1) (Table 1). However, 119 videos from non–health care professionals addressed adverse reactions to removing tattoos, most often pain, blistering, and pigmentation changes; scarring as an adverse event was not mentioned in any of these videos. Among the top 15 most-viewed videos (range 578,340-15,982,270 views), 6 (40%) were created by dermatologists and 1 (7%) by a plastic surgeon. The remaining most-viewed videos were presented by non–health care professionals, none of which encouraged viewers to see a physician for consultation on their tattoo removal. Table 2 summarizes the content of the top 15 most-viewed YouTube videos pertaining to tattoo removal.

**Table 2:** Content of top 15 most-viewed videos in this study.

Rank	Views	Presenter	Tattoo removal method	Does the video recommend seeing a health care professional?	Adverse effects discussed
1	15,982,307	Plastic surgeon	Laser	Yes	Procedural pain
2	10,501,863	Dermatologist	Salt and cocoa butter (and its lack of effectiveness)	Yes	None
3	9,153,031	Patient in tattoo clinic	Laser	No	Procedural pain
4	9,018,118	Dermatologist	Laser	Yes	Procedural pain, bruising, scarring
5	7,879,038	Patient in tattoo clinic	Laser	No	None
6	5,301,535	Dermatologist	Laser	Yes	Procedural pain and swelling
7	1,759,919	Patient in tattoo clinic	Laser	No	Procedural pain
8	1,549,586	Dermatologist	Laser	Yes	Procedural pain
9	1,103,570	Dermatologist	Laser	Yes	Procedural pain
10	1,087,641	Patient at home	Salt scrub	No	Crusting
11	1,035,538	Patient in tattoo clinic	Laser	No	Procedural pain, blistering, pigmentation changes
12	820,358	Patient in tattoo clinic	Laser	No	None
13	758,979	Laser tattoo clinic worker	Laser	No	Pigmentation changes
14	653,883	Dermatologist	Laser	Yes	Procedural pain
15	578,340	Patient at home	Lemon juice and baking soda scrub	No	None

## Discussion

We report that most YouTube videos regarding tattoo removal are presented by nonmedical professionals. While the majority of videos discuss laser-based methods, only a small fraction of videos recommends viewers to visit a dermatology office for these procedures. Because we only analyzed videos presented in English, we were unable to discuss the full breadth of available content to viewers presented in other languages. However, we suspect that similar misinformation patterns exist across languages.

With patients increasingly seeking health information via the internet [7,8], it is important to ensure the provision of high-quality online patient educational materials pertaining to dermatology. Therefore, we suggest patients view YouTube videos on tattoo removal with caution. Dermatologists have tools to address the misinformation that YouTube contains regarding tattoo removal. Beyond contributing to high-quality patient education through YouTube videos on the topic, the major dermatology societies of the country could consider implementing a robust campaign using their social media platforms that encourages patients contemplating tattoo removal to seek consultation with a board-certified dermatologist. In the clinical setting, proactively taking “social media histories” for patients with tattoos who may be contemplating their removal and assessing patients’ understanding of the best way to approach these procedures could be an important opportunity to address areas of misinformation. Ultimately, dermatologists should remain aware of the overall poor quality of information regarding tattoo removal that is publicly accessible on YouTube. Educating patients on how tattoos are safely removed is important to ensure the best cosmetic outcomes while also avoiding potentially serious complications.
